# Formononetin Attenuates IL-1β-Induced Apoptosis and NF-κB Activation in INS-1 Cells

**DOI:** 10.3390/molecules170910052

**Published:** 2012-08-24

**Authors:** Yao Wang, Yunxia Zhu, Lu Gao, Han Yin, Zuoling Xie, Dong Wang, Zhengqiu Zhu, Xiao Han

**Affiliations:** 1 Key Laboratory of Human Functional Genomics of Jiangsu Province, Clinical Diabetes Centre of Jiangsu Province, Nanjing Medical University, Nanjing 210029, Jiangsu, China; 2 Department of Endocrinology, Zhongda Hospital, Institute of Diabetes, Southeast University, No.87 Dingjiaqiao Road, Nanjing 210009, Jiangsu, China

**Keywords:** INS-1, NF-κB, iNOS, formononetin, apoptosis

## Abstract

Several studies suggest that the inflammation plays a role in the pathogenesis of some glucose disorders in adults. Exposure of pancreatic β-cells to cytokines, such as interleukin-1β (IL-1β), is thought to contribute to β-cell apoptosis. One important event triggered by IL-1β is induction of nitric oxide synthase (iNOS), an enzyme that catalyzes intracellular generation of the cytotoxic free radical NO. Recent work have suggested that formononetin, as an *O*-methylated isoflavone found in a number of plants and herbs like *Astragalus membranaceus*, inhibited some pro-inflammatory cytokine production in macrophages. However, the roles of formononetin in pancreatic beta cells have not been fully established. The aim of the present study was to assess possible *in vitro* effects of formononetin on cell apoptosis induced by IL-1β in the rat insulinoma cell line, INS-1. Our results demonstrate that formononetin significantly prevents IL-1β-increased INS-1 cell death and blocks cytokine-induced apoptotic signaling (the reduction of Bax/Bcl-2 ratio and caspase-3 activity). Formononetin also inhibited the activation of nuclear factor-kappaB (NF-κB), which is a significant transcription factor for iNOS, so as to decease nitric oxide (NO) formation in a dose dependent manner *in vitro*. Our observations indicated that formononetin could protect against pancreatic β-cell apoptosis caused by IL-1β and therefore could be used in the future as a new drug improving diabetes mellitus.

## 1. Introduction

It has been well documented that pro-inflammatory cytokines, such as IL-1β, IFN-γ and TNF-α play an important role in the damage of pancreatic β-cells [1,2] by inducing the apoptotic or necrotic destruction of these cells. A recent study has shown that attenuation/prevention of pancreatic β-cell damage induced by inflammatory cytokines is an important step for the treatment of diabetes [3]. Interleukin 1 (IL-1) is a 17 kDa protein highly conserved through evolution and a key mediator of inflammation, fever and the acute-phase responses. IL-1β is produced by macrophages, monocytes, fibroblasts, and dendritic cells, which form an important part of the inflammatory response of the body against infections [4]. IL-1β activates the transcription factor nuclear factor kappa B (NF-κB) and the mitogen-activated protein kinases extracellular signal-regulated kinase, p38 and c-Jun N-terminal kinase [5,6,7]. These transcription factors and mitogen-activated kinases induce the expression of several pro-apoptotic genes, such as Bax, Fas and caspases. Blockade of IL-1β signalling by administration of recombinant IL-1 receptor antagonist or neutralizing monoclonal antibodies has been shown to improve glycemic control in animal models of diabetes [8]. These studies have shown that IL-1 receptor antagonist protected islets from HFD treated animals from β-cell apoptosis, induced β-cell proliferation, and improved glucose-stimulated insulin secretion [9]. Furthermore, treatment of type 2 diabetes patients with recombinant human IL-1 receptor antagonist (Anakinra) resulted in a decrease of glycated haemoglobin levels (a reliable readout for long term glycemia) and improved β-cell function [10].

NF-kappaB (NF-κB) is actually a family of structurally-related proteins that are involved in the control of a large number of normal cellular and body functions, such as immune and inflammatory responses, developmental processes, cellular growth and apoptosis [11]. In addition, NF-κB, as a signal transcription factor, is persistently active in a number of diseases, including cancer, arthritis, chronic inflammation, asthma, neurodegenerative diseases, and heart disease [12,13,14]. The general term NF-κB traditionally refers to the p50/p65 (p50/RelA) heterodimer, which is an apoptotic gene regulator. NF-κB-p65, a subunit of NF-kappaB transcription complex [15], provides the gene regulatory function and plays a crucial role in the development of diseases [16]. The activation of transcription factor NF-κB induced by both TNF-α and IL-1β was associated with the pathogenesis of diabetes mellitus.

NF-κB regulated several genes affected by cytokines in β-cells, including nitric oxide synthase (iNOS), Fas and manganese superoxide dismutase (MnSOD) [17]. During INS-1 cells were exposed to IL-1β, iNOS and Fas contributed to β-cell death were up-regulated, MnSOD was probably involved in β-cell defense against the toxic oxygen free radicals generated [18]. Therefore, NF-κB, as a transcription factor activated by cellular damage and stress, has been thought as a potential pharmacologic target in diabetes mellitus.

Nitric oxide (NO) is now recognized as one of the most important molecules influencing the development, progression and treatment of diseases, which initially described as endothelium-derived relaxation factor (EDRF) in blood vessels. In addition to conveying these physiological processes, excess NO production by a cytokine inducible NOS (iNOS) participates in host defense and immunological reactions and may play a role as effector molecule in autoimmune diseases. Moreover, NO released in response to exposure of pancreatic islets to cytokines may be formed either in β-cells themselves, leading to self-destruction [19]. By binding to the iron-containing Krebs cycle enzyme aconitase that converted citrate to isocitrate, NO inhibited related enzyme activity leading to decrease glucose oxidation, oxygen consumption and adenosine 59-triphosphate (ATP) generation in rat islets [20].

However, the exact molecular signalling mechanisms of NO-mediated rodent beta cell cytotoxicity are unclear. Experiments with human islets have shown that despite an increase in NO production, cytokine-induced human islet functional impairment and cell death cannot be prevented by blockers of NO production [21]. Thus, the exact role and biological significance of NO for human beta cell destruction remain controversial.

Formononetin (biochanin B) is an *O*-methylated isoflavone phytoestrogen from the root of *Astragalus membranaceus*. Several mechanisms have been proposed to explain the *in vitro* anti-inflammatory actions of formononetin, such as antioxidant activity, inhibition of eicosanoid- generating enzymes or the modulation of the production of pro-inflammatory molecules [22]. Flavones significantly suppress inflammation-associated gene expression by blocking NF-kappaB and AP-1 activation pathway in animal models [23,24], but the effect of formononetin on pancreatic beta cells has not been reported. Based on the results described above, the aim of the present study was to investigate the role of formononetin on IL-1β-mediated apoptosis of pancreatic beta cells using the beta cell line INS-1.

## 2. Results

### 2.1. Formononetin (Form) Protects INS-1 Cells from IL-1β-Induced Apoptosis

**Figure 1:**
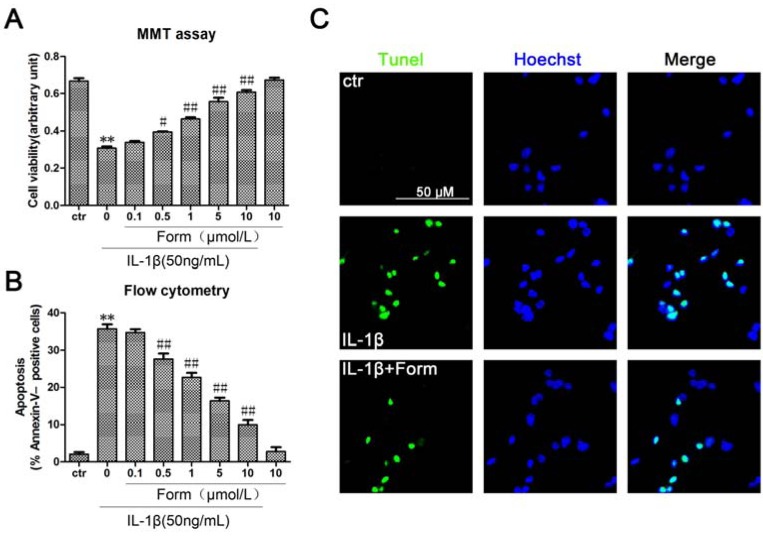
Formononetin inhibited IL-1β-induced apoptosis in INS-1cells. (**A**) Cells were treated with the indicated concentrations of formononetin and IL-1β for 24 h, analyzed by MTT assay. (**B**) Cells werestained with Annexin V-FITC and PI, analyzed by flow cytometry. Data are expressed as % of Annexin V-FITC-positiveand PI-negative cells (early stage of apoptosis). (**C**) Tunel staining of apoptotic cells, IL-1β (50ng/mL) and formononetin (1 μmol/L). Apoptotic cells were marked with green fluorescence, the nuclei of cells are stained by blue fluorescence. Values are the means ± SD (n = 3) of three individual experiments. * *p* < 0.05, ** *p* < 0.01 *vs*. ctr(DMSO); ^#^ *p* < 0.05, ^##^ *p* < 0.01 *vs*. group treated with IL-1β excluding formononetin.

More and more research pinpoints inflammation as a root cause of type 2 diabetes. Chronic exposure to pro-inflammatory cytokines promoting NF-kappaB activation led to apoptosis in β-cells [25]. To investigate the effect of formononetin on the apoptosis of pancreatic β-cells, cell viability was determined in INS cells using MTT assays. Formononetin significant inhibited the decrease of INS cell viability induced by IL-1β in a dose-dependent manner (Figure 1A). The detection of DNA damage by tunel staining indicated that IL-1β decreased the viability and induced apoptosis in INS cells (Figure 1C). Quantitative evaluation of apoptosis through annexin V-FITC/PI staining was analyzed by Flow Cytometry. As shown in Figure 1B, the rate of apoptotic cells were risen to 37.64% with the treatment of IL-1β (50 ng/mL) for 24 h. Furthermore, pretreatment with formononetin prevented IL-1β-induced apoptosis in a dose-dependent manner. Taken together, it suggested that formononetin had a strong anti-apoptotic effect in β-cells.

### 2.2. Formononetin (Form) Suppressed IL-1β-Induced Activation of NF-κB in INS-1 Cells

**Figure 2:**
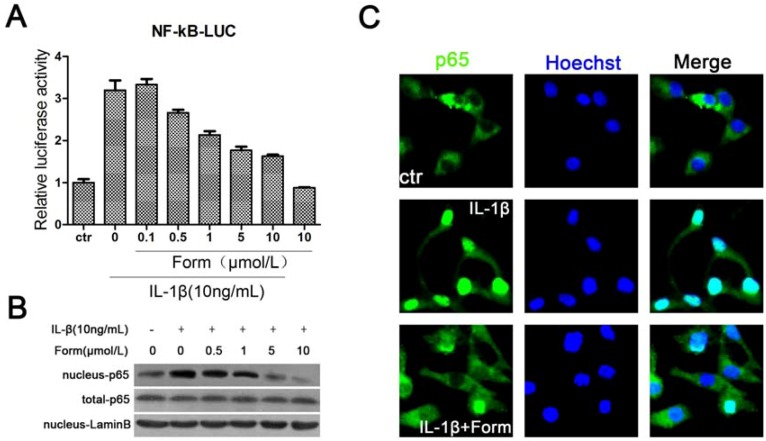
The effect of formononetin on IL-1β-induced activation of NF-κB in INS-1cells. Cells were treated with the indicated concentrations of IL-1β and formononetin for 24h. (**A**) Formononetin dose-dependently reduced NF-κB activation in luciferase reporter assay. Values are the means ± SD (n = 3) of three individual experiments. * *p* < 0.05, ** *p* < 0.01 *vs*. ctr (DMSO); ^#^ *p* < 0.05, ^##^ *p* < 0.01 *vs*. group treated with IL-1β excluding formononetin. (**B**) Protein level of the p65 subunit of NF-κB, as determined by Western blot. (**C**) Representative immunofluorescence localization for p65 subunit. IL-1β increased NF-κB translocation from the cytoplasm to nucleus, IL-1β (50 ng/mL) and formononetin (1 μmol/L). All IL-1β-induced increases were reversed by formononetin.

Nuclear factor kappa B (NF-κB), as a transcription factor, is thought to play an important role in onset of β-cell apoptosis mediated by IL-1β [26]. To determine the effects of formononetin on the inhibition of NF-κB during apoptosis using the luciferase reporter assay, the plasmid (pGL3–3 × NF-κB-Luc) was transiently transfected into INS-1 cells. The results showed that formononetin dose-dependently decreased the level of NF-κB activation induced by IL-1β (Figure 2A). The DNA binding activity was that NF-κB complex was subsequently transported from the cytoplasm to the nucleus and then NF-κB-p65, as a subunit of transcription complex, bound to NF-κB responsive elements [27]. Formononetin inhibited NF-κB complex translocation from the cytoplasm to nucleus, decreasing protein level of nuclear NF-κB (p65) evaluated by Western blot and immunostaining (Figure 2B,C). These data indicated that NF-κB might be a mediator for IL-1β-initiated toxicity in INS-1 cells and IL-1β-induced increases of NF-κB activation were significantly reversed by formononetin.

### 2.3. Formononetin (Form) Inhibited IL-1β-Induced NO Formation and Apoptotic Pathway in INS-1 Cells

**Figure 3:**
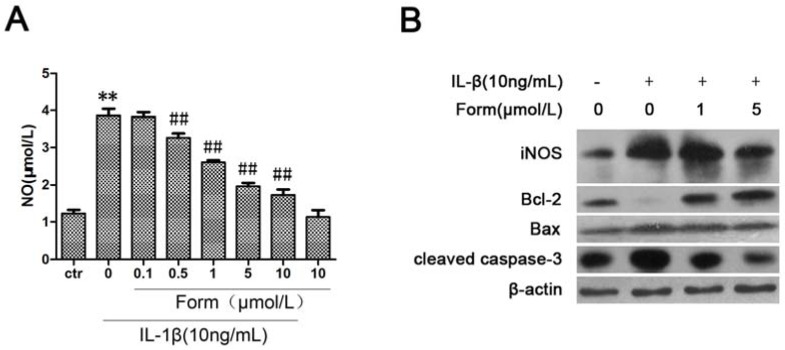
The effect of formononetin on IL-1β-induced NO formation and apoptotic pathway in INS-1 cells. Cells were treated with the indicated concentrations of IL-1β and formononetin for 24h. (**A**) The level of NO in medium of INS-1 cells was determined using Griess method. Values are the means ± SD (n = 3) of three individual experiments. * *p* < 0.05, ** *p* < 0.01 *vs*. ctr (DMSO); ^#^ *p* < 0.05, ^##^ *p* < 0.01 *vs*. group treated with IL-1β excluding formononetin. (**B**) The expression of apoptotic genes were analyzed by western blot in INS-1 cells.

iNOS expression plays a critical role in pro-inflammatory cytokine-induced NO production. Nitric oxide (NO) has emerged as an important endogenous effector of apoptosis [27]. Cytotoxicity as a result of a substantial NO formation is established to initiate apoptosis, characterized by up-regulation of the tumor suppressor p53, changes in the expression of pro- and anti-apoptotic Bcl-2 family members, cytochrome *c* relocation, activation of caspases, chromatin condensation, and DNA fragmentation [28]. In order to determine the effect of formononetin on IL-1β-accumulated NO production by up-regulation of iNOS expression, we investigated the protein levels of iNOS by Western blot and extracellular NO levels from INS-1cells in the media using the Griess method. As shown in Figure 3, iNOS expression and NO production were markedly increased when cells were treated with IL-1β for 24 h. IL-1β caused a change of the expression level of Bcl-2, Bax and caspase-3 proteins as markers of cytokine-induced apoptosis. Moreover, all IL-1β-induced increases were also reversed by formononetin. Therefore, formononetin reduced NO production derived from iNOS induced by IL-1β and inhibited the expression of some apoptotic genes.

## 3. Discussion

More than any other cytokine family, the IL-1 family of ligands and receptors is associated with inflammation. A chronic inflammatory process underlies the failure of the beta-cells to secrete a sufficient amount of insulin in patients with type 2 diabetes [29]. The discrete insulitis is due to a pathological activation of the innate inflammatory/immune system by metabolic stress and is governed by IL-1 signaling [30]. Interleukin-1 beta (IL-1β) also known as catabolin, as one of IL-1 family, plays a major role in IL-1-mediated β-cell dysfunction [31]. Exposure of human islets to glucose, leptin, or free fatty acids (FFA) increases the production and release of IL-1β, hence, therapy targeting IL-1β is a logical choice in protecting the beta-cell in type 2 diabetes [32]. Our results have shown that formononetin, as one of the flavonoids that are commonly present in a large number of vegetables and plants, could effectively inhibit IL-1β-induced apoptosis in INS-1cells. We speculated that NO-dependent apoptosis might be the main toxic pathway for IL-1β in INS-1cells. Apoptosis had been evidenced indirectly by three independent methods in our studies (Figure 1). The expression of proteins in the IL-1β-mediated apoptotic pathway was decreased by formononetin (Figure 3B). NF-κB activation was coincident with the expression of the pro-inflammatory marker iNOS [33]. For this reason, NF-κB might become a candidate target for new anti-inflammatory and anti-apoptosis treatment. Formononetin blocked IL-1β-induced NF-κB activation and reduced NF-κB complex translocation from the cytoplasm to nucleus (Figure 2). Therefore, it suggested that formononetin might be used as a anti-diabetes drug candidate in the following studies.

Diabetes mellitus is a complex disease characterized by absolute insulin deficiency or resistance leading to hyperglycemia. Two major forms of diabetes exist, namely type 1 diabetes mellitus (T1DM) and type 2 diabetes mellitus (T2DM). In T1DM, β-cells are destroyed by immunological mechanisms, whereas in T2DM metabolic abnormalities contribute to β-cell failure and subsequent apoptosis. Regardless of diabetes types, highly conserved intracellular pathways of apoptosis are triggered [34]. Once β-cell mass decreases below a critical level and insulin production no longer meets metabolic demands, hyperglycemia was occurred in patients. The Bcl-2 family and caspase-3 are the major effectors involved in apoptotic pathways [35]. Bcl-2 family has a double-edged effect in diabetes. These proteins are crucial controllers of the mitochondrial pathway of beta-cell apoptosis induced by pro-inflammatory cytokines or lipotoxicity [36]. A common characteristic of cell death after β-cell exposure to pro-inflammatory cytokines or glucolipotoxicity is Bax translocation to the mitochondria, cytochrome c release and activation of the initiator caspase-9, triggering the cleavage of executioner caspases-3. Caspases are evolutionarily conserved cysteine-aspartyl specific proteases that play a key role in cytokine-induced apoptosis in β-cells [37]. Caspase-3-dependent beta-cell apoptosis was observed in diabetic animals, such as db/db and NOD mice [38,39]. Together, our experimental models have shown the importance of IL-1β-induced β-cell apoptosis.

Bio-flavonoids comprise a group of phenolic secondary plant metabolites that are widespread in Nature. A good number of studies have already demonstrated many biological activities of flavonoids using different experimental models and treatments. Formononetin (biochanin B), is a major phytoestrogen found in alfalfa and clover sprouts, that has been reported to have beneficial effects for Alzheimer's disease (AD) and anti-oxidant properties [40,41]. The effect of formononetin on metabolic diseases is still unknown, but biochanin A, as an analog of formononetin, improved impaired glucose tolerance and protected against body weight loss of streptozotocin-diabetic animals [42]. Biochanin A, also an isoflavone, showed anti-inflammatory activities through the inhibition of iNOS expression, p38-MAPK and ATF-2 phosphorylation and blocking NF-κB nuclear translocation [43]. For this reason, we have attempted to choose some flavonoids as target compounds for our studies and formononetin shown a strong anti-apoptosis effect.

## 4. Experimental

### 4.1. Reagents

Formononetin (HLPC content 98%) was purchased from Shanghai Winherb Medical S & T Development Co. Ltd (Shanghai, China) RPMI-1460 medium and fetal bovine serum (FBS) were purchased from Gibco (Grand Island, NY, USA). The lipofectamine 2000 reagent was obtained from Invitrogen Life Technologies (Grand Island, NY, USA). The luciferase reporter assay system and avian myeloblastosis virus reverse transcription system were obtained from Promega (San Luis Obispo, CA, USA). The rabbit polyclonal antibody against iNOS was purchased from Santa Cruz Biotechnology (Delaware Avenue, CA, USA). The rabbit polyclonal antibodies against p65, Bcl-2, Bax and caspase-3 were purchased from Cell Signal Technology (Boston, MA, USA). Nitric Oxide Assay Kit (Griess Reagent) was purchased from Beyotime (Haimen, China). The Annexin-V-FITC Apoptosis Detection Kit was purchased from BD Biosciences (San Jose, CA, USA). Terminal Transferase dUTP Nick End Labeling (TUNEL) Assay kit was purchased from Roche (Boston, MA, USA).

### 4.2. Cell Culture

INS-1, a rat insulinoma cell line, was obtained from the American Type Culture Collection (ATCC, Manassas, VA, USA). The cells were grown in RPMI-1640 medium supplemented with 10% fetal bovine serum (FBS), 10 mmol/L HEPES, 2 mmol/L L-glutamine, 50 μmol/L β-mercaptoethanol, 1 mmol/L sodium pyruvate, 100 U/mL penicillin, and 100 μg/mL streptomycin at 37 °C in a humidified atmosphere containing 95% air and 5% CO_2_. The cells were exposed to various concentrations (0.1–10 μmol/L) of formononetin or to IL-1β (10 ng/mL or 50 ng/mL) for the indicated times. Before the co-treatment with IL-1β and formononetin, cells were pretreated with formononetin for 2 h.

### 4.3. MTT Assay

Cell viability was determined using the 3-(4,5-dimethylthiazol-2-yl)-2,5-diphenyltetrazolium bromide (MTT) assay. Briefly, the cells were seeded in 96-well dishes at 1 × 10^4^ to 2 × 10^4^ cells per well, and pretreated with or without IL-1β for 24 h. Each well was then supplemented with 10 μL MTT (Sigma) and incubated for 4 h at 37 °C. The medium was then removed, and 150 μL dimethyl sulfoxide (Sigma) were added to solubilize the MTT formazan. The optical density was read at 490 nm.

### 4.4. Flow Cytometry

To estimate the number of apoptotic cells, cells were fluorescently labeled by addition of 20 μL of binding buffer, 5 μL of Annexin V-FITC and 5 μL of propidium iodide. After incubation at room temperature in the dark for 15 min, cells were subjected to flow cytometry analysis. A minimum of 10,000 cells in the gated region was analyzed by a BD FACS Calibur Flow Cytometer. Results were interpreted by the percentage of total cells appearing in each quadrant.

### 4.5. Tunel Staining

Cells were cultured on coverglasses in 12-well plates. After 24 h treatment, the apoptotic cells were stained by tunel staining kit following its protocol, the apoptotic cells were stained by green fluorescence, and all cells were marked with blue fluorescence using DAPI. The apoptotic ratio was calculated as tunnel-positive cells divided by total cell number. The number of cells was counted in three random fields from three different slides at 200× magnification. An average for the percentage of tunnel-positive cells was taken over these fields. The sections were observed by two pathologists in a blinded manner.

### 4.6. Immunofluorescence Microscopy

A standard immunostaining procedure was carried out to observe NF-κB nuclear translocation activity. Cells grown on thick slides were washed with PBS, fixed by immersion at room temperature with 4% polyformaldehyde for 20 min, and permeabilized with 0.1% Triton-X-100 in PBS at 4 °C for 10 min. Slides were then washed with PBS and blocked with blocking buffer consisting of 4% bovine serum albumin (BSA) in PBS for 30 min at room temperature and then incubated with primary anti-NF-κB (p65) monoclonal antibody diluted 1:25 in blocking buffer overnight at 4 °C, followed by a secondary anti-rabbit FITC-labeled antibody incubation diluted 1:100 in blocking buffer at room temperature for 1 h. Subsequently, cells were stained with 5 μg/mL hoechst for 2 min and washed with PBS. Coverslips and stained cells were captured and analyzed by a confocal laser scanning microscopy system using a 400× magnification (LSM710, Carl Zeiss, Heidelberg, Germany). Control samples with HRP-conjugated secondary antibody and no DAPI showed a faint background staining (data not shown). Numbers of NF-κB (p65) translocated into the nuclei were labeled by examining 50 cells per-random field from one slide under a fluorescence microscope.

### 4.7. Transient Transfection and Luciferase Reporter Assay

The luciferase reporter construct 3 × NF-κB-LUC (NF-κB responsive elements) was transiently transfected into INS-1 cells grown in 24 well plates respectively using the lipofectamine 2000 reagent according to the manufacturer’s instructions. The luciferase reporters construct driven by three copies of the NF-κB response elements from B.M. Forman (Department of Gene Regulation and Drug Discovery, Beckman Research Institute of City of Hope National Medical Center, Duarte, CA, USA). A plasmid expressing the gene encoding β-galactosidase driven by the cytomegalovirus (CMV) promoter (Clontech Laboratories, Palo Alto, CA, USA) was simultaneously cotransfected as an internal control. The medium was replaced 4 h after transfection. Twenty-four hours after transfection, the cells were treated with the indicated concentrations of IL-1β and formononetin for an additional 24 h and harvested for luciferase reporter assays as described previously [44].

### 4.8. Western Blot

Cells cultured and treated as described above, and the lysed with ice-cold lysis buffer containing50 mmol/L Tris-HCl, pH 7.4; 1% NP-40; 150 mmol/L NaCl; 1 mmol/L EDTA; 1 mmol/L phenylmethylsulphonyl fluoride; and complete proteinase inhibitor mixture (one tablet per 10 mL; Roche Molecular Biochemicals, Indianapolis, IN, USA). After protein content determination using a DC Protein Assay kit (Bio-Rad Laboratories, Hercules, CA, USA), Western blot was performed as described previously [45].

### 4.9. NO Assay

INS-1 cells were seeded into 48-well plates for 24 h, and Media subsequently were replaced by 200 μL of serum-free medium per well with the indicated concentrations of IL-1β and formononetin. After 24 h of incubation, the medium was sampled for NO determination using the Griess method. 

### 4.10. Statistical Analysis

Differences between groups were analyzed using two-sided t test and ANOVA with *p* < 0.05 considered statistically significant.

## 5. Conclusions

We have shown that formononetin could inhibit IL-1β-induced NF-κB activation and β-cell apoptosis. This molecule may have further utility in clinical applications for treating diabetes mellitus in the future.
